# Improving the diagnostic accuracy of referrals for papilloedema (DIPP) study: protocol for a mixed-methods study

**DOI:** 10.1136/bmjopen-2024-090521

**Published:** 2025-01-28

**Authors:** Alyson Huntley, Olivia Skrobot, Matthew J Ridd, Mary-Ann Sherratt, Marcia Lucraft, Christina Stokes, Michael Bowen, Beth Stuart, Samuel William David Merriel, Denize Atan, DIPP group, Victoria Wilson

**Affiliations:** 1 Centre of Academic Primary Care, University of Bristol, Bristol, UK; 2 University of the West of England, Bristol, UK; 3 Bristol Eye Hospital, University Hospitals Bristol & Weston NHS Foundation Trust, Bristol, UK; 4 Director of Research, The College of Optometrists, London, UK; 5 Wolfson Institute of Population Health, Queen Mary University of London, London, London, UK; 6 Centre for Primary Care & Health Services Research, The University of Manchester, Manchester, UK; 7 Translational Health Sciences, Bristol Medical School, University of Bristol, Bristol, UK

**Keywords:** Surveys and Questionnaires, QUALITATIVE RESEARCH, OPHTHALMOLOGY, Primary Health Care

## Abstract

**Abstract:**

**Introduction:**

Papilloedema can be the first sign of life-threatening disease, for example, brain tumours. Due to the potential seriousness of this clinical sign, the detection of papilloedema would normally prompt urgent hospital referral for further investigation. The problem is that many benign structural variations of optic nerve anatomy can be mistaken for papilloedema, so-called pseudopapilloedema. The consequence is that many people are referred to hospital because they are incorrectly identified to have papilloedema when they don’t. As a result, hospital referrals of people with suspected papilloedema in England have increased sharply, leading to increased demand for overstretched hospital services and potentially longer waiting times for hospital appointments for those who do have papilloedema.

The work programme is aimed at the development of guidelines and educational materials that will help support health professionals to correctly identify people with papilloedema. This article describes the protocol for gathering evidence of current referral practices and pathways for people suspected to have papilloedema in England and the development of guidelines based on this evidence and extensive engagement with community- and hospital-based healthcare professionals, patients, and the public.

**Methods and analysis:**

Both qualitative and quantitative data will be collected from Freedom of Information requests to Integrated Care Boards across England about how they organise their community and hospital services for people with suspected papilloedema, with and without headache. Surveys and qualitative interviews of relevant community and hospital healthcare professionals based in England will collect data on how and when people with papilloedema and pseudopapilloedema with or without headache are currently identified and referred to hospital, if needed. This information will be used to inform a Delphi process with the aim of reaching consensus among health professional experts, commissioners and patients on what the most evidence-based and safe diagnostic and referral practices should be for people with suspected papilloedema. The tailored guidelines will be written for healthcare professionals and patients. We will create a range of educational materials and a website designed for health professionals and patients to support the national roll-out and implementation of DIPP study guidelines.

**Ethics and dissemination:**

Ethical approval was granted by the University of Bristol Faculty of Health Sciences Ethics committee (FREC reference: 12457) and Health Research Authority (IRAS no.: 320395). Results of the study will be published on our DIPP study website and disseminated to our stakeholder groups through peer-reviewed journal publications and conference presentations.

**STRENGTHS AND LIMITATIONS OF THIS STUDY**

- It uses established survey, qualitative and Delphi methodology.
- It involves engagement/consultation with professional stakeholders.
- It works with patient and public members with relevant lived experiences.
- It focuses on services in England relevant to primary and secondary care.
- Its outputs will allow for the variability of community eye services in England.

## Introduction

 Papilloedema refers to swelling of the optic nerve head, at the back of the eye, due to raised intracranial pressure (ICP). The importance of this clinical sign is that papilloedema can be the first sign of life-threatening disease, for example, brain tumours; hence, the detection of papilloedema usually prompts urgent hospital investigations.^1^ In some cases, general practitioners (GPs) and other front-line primary care professionals may be able to request urgent brain scans for patients with visual symptoms, headache and/or papilloedema, suggestive of raised ICP. However, headaches are common among the population and the positive predictive value of headache for a brain tumour is only 0.2%.^2^ Furthermore, GPs are likely to have had less clinical and skills training to detect papilloedema than eye specialists and may lack the correct equipment to do so. Consequently, GPs often recommend patients with visual symptoms/headaches to have a National Health Service (NHS) sight test by an optometrist trained to detect problems with vision and general health and would then refer these patients to secondary care if papilloedema is suspected. Equally the public may first choose to consult with a community optometrist about their visual symptoms and/or headaches, and if papilloedema is suspected, the optometrist would write to their GP to refer them to hospital. As a result, optometrists may be the first health professionals to suspect papilloedema in people sent to them by their GPs or attending their practices for routine eye tests.

In principle, community optometrists have the skills and equipment to diagnose papilloedema. Severe papilloedema is not difficult to recognise, but it takes experience and training to distinguish mild papilloedema from conditions that can mimic it (pseudopapilloedema) as neither pseudopapilloedema nor mild papilloedema usually causes symptoms. For example, ‘crowded’ optic nerves in longsighted people and ‘drusen’ (benign protein deposits) buried within the optic nerves are causes of pseudopapilloedema but are not serious threats to health or vision and do not usually require hospital referral. Specialist imaging tests like optical coherence tomography (OCT), autofluorescence imaging and ultrasound can help distinguish papilloedema from pseudopapilloedema, but community optometrists vary in the equipment they have available, and their training to interpret the results, which in one case led to a legal case of gross negligence (although this conviction was later quashed).^3 4^ Consequently, some optometrists have become more anxious about missing papilloedema and less confident about distinguishing mild papilloedema from pseudopapilloedema. As a result, hospital referrals of people suspected to have papilloedema in England have increased sharply, leading to increased demand for overstretched hospital services.^5^

This increased demand for secondary care services nationally, as well as hospital eye services, has led to more brain scan requests.^6^ People with pseudopapilloedema who are referred to hospital might be told ‘they could have a brain tumour’, causing undue stress and anxiety while they wait for appointments. On the other hand, patients who do have papilloedema might have to wait longer for appointments.

In the absence of nationally adopted guidelines for the diagnosis and referral of patients with papilloedema versus pseudopapilloedema, and because NHS service provision and resources vary between hospitals and primary care services across the country, it is likely that these patients are managed very differently from region to region.

The three objectives of the DIPP study are as follows:

**Objective 1:** to gather evidence of current referral practices and pathways for people suspected to have papilloedema/ pseudopapilloedema in England.

The related study research questions are:

- Which community and hospital services and referral pathways currently exist for people with suspected papilloedema/ pseudopapilloedema?
- How are people with papilloedema/pseudopapilloedema currently diagnosed, and which investigations are used by community- and hospital-based health professionals?
- Which factors influence decisions by GPs and optometrists to refer people with papilloedema/pseudopapilloedema to hospital for further investigations? Are there local referral criteria that influence these decisions?

**Objective 2:** to develop best evidence and consensus-based guidelines and supporting educational materials to improve patient safety and quality of care, and that are adaptable to available resources across England.

The related study research questions are:

- Is there consensus among health professionals, commissioners and patients on how we can improve diagnostic and referral pathways for patients with papilloedema/ pseudopapilloedema?
- What information is needed by health professionals nationally to help them understand, use and keep up to date with the diagnostic accuracy of referrals for papilloedema (DIPP) study guidelines?

**Objective 3:** to prepare for a national roll-out and implementation of our proposed DIPP study guidelines through dissemination, a range of educational materials and a website for health professionals. Part of these educational materials and the website will provide patient-friendly information, which can also facilitate health professional support for patients.

## Methods and analysis

### Study design

The DIPP project is a mixed-methods study that aims to improve the diagnostic accuracy of referrals for papilloedema versus pseudopapilloedema from primary to secondary care. The study commenced in May 2022 and has a planned end date of March 2025.

### Project team

#### Study team

The team who will conduct and/or advise on the work detailed below comprise researchers, medical students, clinicians and representatives from key stakeholder groups (patients and public, optometry, GP, ophthalmology, neurology, emergency medicine).

The DIPP group also comprises a statistician, health economist, PPIE advisor, communications manager, and members from the Bristol, North Somerset, South Gloucestershire Integrated Care Board, the Avon Local Optical committee Chair, the College of Optometrists and the National Service Delivery Manager for Specsavers opticians.

These relationships will help ensure our research outputs reach their full potential in their development, implementation and evaluation.

#### Patient and public involvement

The DIPP study has two patient and public involvement and engagement (PPIE) groups: a hospital PPIE group (six patients diagnosed with brain tumours) and a community PPIE group (six members of the public who use community optician services). The hospital and community PPIE groups have chosen to remain separate to allow full uninhibited expression of their opinions. We have endeavoured to get a representative mixture of ages, ethnicity and gender. We will work with our PPIE groups throughout the DIPP project to ensure our patient-facing materials are clear and appropriately worded. One PPIE member from the hospital PPIE group and one from the community PPIE group have agreed to work closely with us attending key research meetings. Contact with our PPIE groups will be varied based on the contributors’ preferences. We will offer contact via video conferencing, post, telephone and email.

### Methods

**Objective 1:** to gather evidence about how different health professionals and NHS services usually manage people with suspected papilloedema, with and without headache, across England.

#### Freedom of Information requests to Integrated Care Boards

We will devise a set of questions to find out how Integrated Care Boards (ICBs) organise their community and hospital services for people with suspected papilloedema with and without headache, suggestive of raised ICP. Since hospital referrals may not distinguish between papilloedema and pseudopapilloedema and may instead refer to ‘blurred disc margins’, ‘raised disc margins’, ‘indistinct disc margins’ or ‘optic disc swelling’, we will include these terms in our request. The questions will be piloted with two ICBs to check they obtain appropriate data before sending to all remaining ICBs in England via email using the formal Freedom of Information (FOI) process. Responses to FOI requests are currently required within 20 working days, so we expect a 100% response to these requests.

#### Online surveys of primary and secondary care health professionals

Optometrists, GPs, ophthalmologists, neurologists and emergency care clinicians are the primary and secondary care health professionals who are typically expected to diagnose and manage patients with suspected papilloedema with and without headache. The aim of the surveys is to determine the factors that influence the decision-making of these health professionals from different clinical backgrounds, knowledge and training when they are presented with very similar clinical scenarios. We will survey these health professionals using questions based around a series of case vignettes or clinical scenarios that describe patients with suspected papilloedema/pseudopapilloedema. Although the case vignettes will be similar across all the health professional surveys, the questions are tailored to each professional group being surveyed as to the contexts in which they might encounter these cases, for example, in their optician practice or the emergency department of a hospital (Online supplemental appendix 1). The surveys will ask questions about each clinician’s approach to the investigation, diagnosis and referral of these patients. Questions about the information they would normally provide to patients will be devised with our PPIE groups.

The participant information sheets about the study, consent forms and the surveys will be administered using Research Data Capture (REDCap), a secure online repository for research surveys managed by the University of Bristol IT services.^7 8^ They will be piloted by the relevant health professionals within our study team and colleagues, prior to dissemination by email. We will ask the different professional groups and other health networks to help us disseminate the surveys. These groups and networks will include the College of Optometrists, Royal College of Ophthalmologists, Royal College of Emergency Medicine, Association of British Neurologists and UK Neuro-ophthalmology special interest group to ensure representation of eye care professions. We will not be requesting mailing lists but will ask these professional groups and networks to send out a recruitment email on our behalf. We will also recruit via our local National Institute of Health and Care Research (NIHR) Clinical Research Networks (CRNs) who can reach out to the other national CRNs. This will allow us to recruit GPs. The email will include a brief outline of the study with links to the DIPP study website. We will also use recruitment adverts that can be posted on websites, newsletters and social media platforms. We will monitor geographical representation as we receive participation. We are not able to define a response rate for these surveys as we cannot enumerate the population we will reach, but our approach is to be inclusive and get good regional variation in the responses.

Participants who agree to take part in the survey will have the opportunity to provide their contact details on the consent form if they would like to be invited for interview or later partake in the Delphi process part of the study.

Participation is entirely voluntary, and even participants who provide their contact details in the consent form can opt out of the interviews and Delphi process when approached later in the study.

After completing the electronic consent form, the participant will be redirected to the online survey for their profession. Survey responses will be stored separately from the consent forms in REDCap but linked using a pseudo-anonymous ID generated using the REDCap software. To avoid the collection of personal or identifiable information in the surveys, respondents will be asked about the approximate geographical region where they work, and some characteristics of their workplace (eg, multiple or independent optometry practice) but not their name, home address, date of birth, specific practice or hospital. The surveys will be kept to a maximum time of 20 min to complete to ensure greater uptake.

#### In-depth interviews of health professionals from different regions of the UK

We will invite primary and secondary care practitioners to interview those who consented to provide their details when they completed their consent forms for the surveys. We will use purposive sampling to ensure we capture the views of health professionals who serve different geographical areas and populations in England, for example, rural versus highly populated areas, that may differ in referral processes. Up to 30 semistructured interviews (of up to 30 min each) will be conducted with up to 10 optometrists, 10 ophthalmologists and 10 GPs. We will stop conducting interviews when we agree as a group that we have reached saturation in the ideas expressed by each health professional group interviewed. We have not included neurologists in the interview schedule as we felt they are at the specialist end of the pathway and interviews would not provide us with sufficient relevant information for our guidance. The interviews will be audio-recorded and conducted by video call. The results of the relevant health professional survey and FOI data will be used as topic guides. This will provide more depth to our understanding of geographical variations in current practices, although we cannot guarantee data saturation within these numbers.

#### Synthesis of data from FOIs, surveys and interviews

FOI responses will be collated in a customised Excel spreadsheet for framework analysis. The results of surveys and interviews will be collated and written up using narrative summary and thematic analysis. Quantitative evidence from the survey questions regarding the case vignettes (eg, diagnosis and referral destinations) and information about current practice, for example, availability of local protocols, and referral trends will be summarised using descriptive statistics. Summary tables will be produced from which we will identify areas of need and geographical differences. We will work with our PPIE groups to co-produce plain English summaries of these results.

**Objective 2:** to reach consensus among health professional experts, commissioners and patients on what the most evidence-based and safe diagnostic and referral practices should be for people with papilloedema/pseudopapilloedema across England.

#### Delphi process

The Delphi technique is an established method for achieving consensus, particularly where there is no existing agreement or where contention is expected.^9^ This technique has both strengths and limitations. We have based our questions on published international evidence, contextualised to service provision in England to ensure the relevance of the content. We have been clear about how we are defining consensus, a common criticism of Delphi processes. It is, of course, a complex and time-consuming process that is subject to participants changing their minds between rounds and dropouts. We are committed to recording our responses and presenting our data with appropriate caveats.

We anticipate that current practices will vary depending on geographical location and variation in local primary eye care provision, expertise, equipment and technology, and availability of specialist hospital services, like neurology and ophthalmology.

We will apply a modified Delphi process, comprising 2–3 rounds and a final consensus meeting. The final face-to-face meeting was not a component of the original Delphi method; it was adopted from the modified Ebel procedure^10^ and is also known as the Estimate-Talk-Estimate process.^11^

Participants in our Delphi process will be recruited from the optometrists (with ≥5 years’ experience according to the General Optical Council public register: https://str.optical.org/), GPs, ophthalmologists, neurologists and emergency care clinicians across England who agree to be contacted to participate in our Delphi process in the consent form to the survey. In addition, we will use recruitment adverts that can be posted on websites, newsletters and social media platforms. Our participant pool will include at least 50 healthcare professionals. We will use purposive sampling to ensure we capture the expert opinions of health professionals from across England by inviting those with a reputation/publication record in this area through the support of our research team who have expertise in these specialties and contacts within national networks and professional bodies.

In addition, the College of Optometrists works with a nationally recruited PPIE group who we will approach through one of our co-applicants. Volunteers from this national group will be asked to participate in the working group that oversees our Delphi process to eliminate local biases and add their perspectives.

The modified Delphi process will have 2–3 rounds conducted through online surveys, followed by a virtual consensus meeting. Participants will be provided with an information sheet and asked for their electronic consent to take part in our Delphi process, administered using REDCap. They will be provided with knowledge summaries of the relevant literature as they complete the Delphi survey, including the results of a systematic review and meta-analysis currently under peer review entitled *Optic Nerve Imaging to Distinguish Papilloedema from Pseudopapilloedema: A Systematic Review and Meta-Analysis* (PROSPERO [CRD42020213223]).

Their responses to the Delphi surveys will be stored separately from their consent forms but linked by an ID generated in REDCap that ensures they are pseudo-anonymous. As with the health professional surveys of current practice described above, we will avoid the collection of personal or identifiable information about respondents who will be asked about the approximate geographical region where they work and some characteristics of their workplace (eg, multiple or independent optometry practice) but not their name, home address, date of birth, specific practice or hospital. The surveys are designed to take a suggested time of 20 min to complete to avoid attrition between survey rounds. They will be piloted to health professional colleagues who are not participants in our Delphi process before wider dissemination for the study.

Participants in the Delphi surveys will be sent emails reminding them of the survey deadline for each survey round. Participants will be asked to save a copy of their responses after completing each round, when prompted in REDCap, to compare with the overall results that are provided in the subsequent round.

Each round of the modified Delphi process will be as follows:

**Round 1**: Participants will be asked to rank the importance of features in the clinical history, examination and relevant investigation results that influence their decision to refer patients with papilloedema or pseudopapilloedema to secondary care on a 9-point scale. These items will be derived from published literature and the responses to our FOI requests, and health professional surveys and interviews. Participants will be given the opportunity to skip questions that fall outside their specific area of clinical expertise. For example, we do not anticipate GPs will answer questions about specialist ocular imaging, such as OCT.

Data will be exported from REDCap for descriptive analysis in Microsoft Excel (eg, median, percentages). Free-text responses will be narratively summarised. Elements that participants propose as important for consideration that were not previously included, will be surveyed in the next stage of the process.

**Definition of consensus**: Features will be considered important if the median score is at least 7 on the 9-point scale. Features with median scores between 4 and 6.9 will be considered uncertain and participants will be asked to judge these features again. Features will be rejected if ≥70% of participants judge them as ‘not appropriate’ or if the median score is ≤3.9. All other features will be taken through to the consensus meeting where statements will be presented to participants and asked if they agree or disagree (vote yes or no). Agreement will be considered if ≥70% of participants vote the same (yes, accepted; no rejected).
**Round 2**: Will repeat the above process with reporting of the results of round 1.
**Round 3**: Participants will select the clinical features that influence the urgency and destination of their referrals of patients with papilloedema or pseudopapilloedema. They will also be asked which service they think is most appropriate to refer patients depending on the suspected diagnosis and urgency of the referral. Participants will also comment on the information that ought to be shared between health professionals for referrals to secondary care to work most effectively. They will be asked what information ought to be shared with patients to ensure they receive the information they need without causing undue anxiety.
**Consensus meeting**: Ranking of features in the previous 2–3 rounds of the modified Delphi, together with the evidence we have gathered from the published literature, FOIs, surveys and interviews, will be reviewed in a virtual consensus meeting to which participants and PPIE contributors of the Delphi will be invited. Unresolved features from the Delphi and disagreements during the consensus meeting will be reviewed by attendees who will be presented with the relevant information. The participants will then be asked to respond, ‘yes’ or ‘no’ to a ‘consensus question’. If there is no agreement, discussion about phrasing of these statements can take place that may lead to slightly different wording of the statement that is then put forward again for a vote. If any outcome has a majority of ‘yes’ responses after this process, it will be accepted and included in our guidelines for recommended practice. In this meeting, we shall also consider the resource and health economic cost implications of our guidelines.

#### Drafting of DIPP guidelines and external review

The anticipated output of our Delphi process will be agreement on written DIPP guidelines that are clear, precise and actionable. They will be tailored to the relevant health professional group (GPs, optometrists, ophthalmologists, neurologists, emergency care clinicians) following the Reporting Tool for Practice Guidelines in Health Care (RIGHT) checklist for clinicians.^12^ Additional versions of DIPP guidelines will be tailored to patients following the RIGHT checklist for patient versions of guidelines, RIGHT-PVG.^13^ Additionally, we will prepare a descriptive analysis of relevant health professional and patient opinions, any quantitative data that arises and a summary of the resource and cost implications of the guidelines.

**Objective 3:** to prepare for a national roll-out and implementation of our proposed DIPP study guidelines through dissemination, a range of educational materials and a website for health professionals. Part of these educational materials and website will be patient-friendly information which can also facilitate health professional support for patients.

#### Communication goals

(1) To make eye care and other health professionals and GPs aware of the study and availability of DIPP guidelines and training; (2) to inform patients and the public about what the study means for them; (3) to make policy and commissioning audiences, for example, NHS England, aware of the guidelines and training to improve the quality and cost-effectiveness of patient care; and (4) to gain support among funders and the academic community for further dissemination and implementation work.

#### DIPP study website

This is hosted by the University of Bristol and will be updated throughout the project. The final guidelines and educational resources will have ‘DIPP study’ branding and will be available through the website (https://www.bristol.ac.uk/primaryhealthcare/researchthemes/dipp-study/)

#### Production of resources

We will initially prioritise the production of educational resources that are tailored toward optometrists; we will then adapt the materials to suit other stakeholders (GPs, ophthalmologists, neurologists, emergency care clinicians, public).

## Ethics and dissemination

### Dissemination

#### Objective 1: evidence gathering

We will synthesise and summarise qualitative and quantitative data from the FOIs, surveys, interviews and literature reviews. Suitable quantitative evidence will be summarised using descriptive statistics. We will work with our PPIE group to co-produce plain English summaries of these results. The results of this evidence synthesis will be presented at national conferences and published in peer-reviewed publications.

#### Objective 2: Delphi process

Summaries from our evidence synthesis will then be used to inform our Delphi process. The Delphi will result in qualitative outputs: guidelines for recommended practice and educational materials. We plan to publish our guidelines and educational materials on the DIPP study website and disseminate them through conference presentations and peer-reviewed publications.

#### Objective 3: supporting national rollout and implementation

These educational materials will include a mixture of prerecorded lectures, live and interactive webinars (using the latest webinar platform available through the University of Bristol) and online content (decision-tools, infographics, patient information) that are tailored to users of the guidelines (optometrists, GPs, ophthalmologists, neurologists, emergency care clinicians) and can be downloaded or printed from the website. The patient-facing materials will be co-produced with our community- and hospital PPIE groups, College of Optometrists' Patients and Public Reference PPIE group. These will include printed, online and audiovisual patient-friendly outputs that will be available (downloadable) on the website, including Patient Information leaflets explaining ‘what to expect’ following referral to hospital; plain English summaries and infographics, with EASY READ15 versions and (potentially) translations to other languages (https://www.easy-read-online.co.uk/).

### Ethics

Ethical approval was granted by the University of Bristol’s Faculty of Health Sciences Ethics committee (FREC reference: 12457) and Health Research Authority (IRAS no.: 320395).

### Legal issues

Participants will be asked to provide their electronic consent before completing the surveys in REDCap. Survey responses will be pseudo-anonymous as they will be stored separately from the consent information but linked using an ID generated in REDCap.

Participants will be asked for their additional consent to provide their personal details (name and email address) so that we might contact them to arrange an interview with them and/or ask for their participation in the Delphi process. Participants will be asked again for their consent to record their interview and/or record the Delphi consensus meeting. Recordings will be deleted once the audio has been transcribed. The recordings will not be used for any other purpose than the study. We will use pseudonyms for stored data where necessary. No one other than the members of the research team will have access to these data.

### Data management issues

Anonymised data and consent forms will be stored within the University of Bristol’s Research Data Storage Facility. The University of Bristol (organisation code EE133799-BRMS) has a Data Storage and Protection Toolkit and meets 2019/2020 Data Security Standards.

### Archiving

We will keep anonymised data for up to 3 years following study completion to ensure all statistical analyses are completed, available through peer review and published. We will store personal data and consent forms for up to 12 months after the study has ended. We will then dispose of the information in accordance with General Data Protection Regulation (GDPR). Anonymised information will be kept in secure password-protected and encrypted data storage that is only accessible to individuals in the research team. Any adverse events (AEs) will be reported to the University Hospitals Bristol and Weston NHS Foundation Trust’s Research and Development team, which handles the University of Bristol’s AE reporting under a Service-Level Agreement.

## supplementary material

10.1136/bmjopen-2024-090521 online supplemental file 1
