# Supplementary material for: Improving the diagnostic accuracy of referrals for papilloedema (DIPP) study: protocol for a mixed-methods study
Source: BMJ Open. 2025 Jan 28;15(1):e090521. doi: 10.1136/bmjopen-2024-090521 (PMC11781146; doi:10.1136/bmjopen-2024-090521)
Supplement: online supplemental file 1 [file bmjopen-15-1-s001.docx]

**Appendix 1: Case vignettes**

**Case 1:** A 25-year-old woman presents to [your practice*] with a history of headaches for the last 4 months. She is overweight with a BMI over 30. She has no past medical history and does not take any medication or analgesia for her headaches.

*context changes for each professional

Colour fundal images of tilted and crowded optic discs are presented beneath this description.

**Case 2** (optometrists survey): A 32-year-old man comes to your practice for a routine eye test. He says he is fit and healthy, but his prescription needs updating.

Colour fundal images of papilloedema are presented beneath the description

The context for Case 2 for the other professionals is a referral letter from the optometrist:

Case 2: You receive a referral letter from a local optometrist about a 32-year-old man whom they have seen for a routine eye test. The patient went to see the optician because they thought their prescription needed to be updated, which it did. He is normally fit and healthy so no-one at the GP surgery has seen the patient for some time. He does not take medications. The optometrist has examined his fundi and says, in the referral letter, that the optic disc margins look raised, blurred, and indistinct.
