# Supplementary material for: Improving the diagnostic accuracy of referrals for papilloedema (DIPP) study: protocol for a mixed-methods study
Source: BMJ Open. 2025 Jan 28;15(1):e090521. doi: 10.1136/bmjopen-2024-090521 (PMC11781146; doi:10.1136/bmjopen-2024-090521)
Supplement: Supplementary file 2 [file bmjopen-2024-090521.reviewer_comments.pdf]

PEER REVIEW HISTORY

BMJ Open publishes all reviews undertaken for accepted manuscripts. Reviewers are asked to complete a checklist review form and are provided with free text boxes to elaborate on their assessment. These free text comments are reproduced below.

ARTICLE DETAILS

Title (Provisional)

Improving the Diagnostic accuracy of referrals for Papilloedema (DIPP) study: protocol for a mixed methods study.

Authors

Huntley, Alyson; Skrobot, Olivia; Ridd, MJ; MA, Sherrat; Lucraft, M; Stokes, C; Bowen, Michael; Stuart, Beth; Merriel, Samuel William David; Atan, Denize

---

VERSION 1 - REVIEW

**Reviewer**	**1**
**Name**	**Milea, Dan**
**Affiliation**	**Singapore National Eye Centre, Singapore,**
**Date**	**12-Aug-2024**
**COI**	**N/A**

In the proposed article, the authors describe very thoroughly their planned research on referrals for papilledema (and its differential diagnosis). There is a high need for such a study and the authors have produced a proposal which very well conceived, most likely due to the collaborative participation of experts from multiple fields (GPs, ED doctors, ophthalmologists, neurologists, statisticians, population health experts, etc). I have only a few, very limited remarks

- I do not see the dates of the planned study in the mansucript

- Why is this study limited to England and does not include the whole UK, as other major national studies have done before? Having the paritcipation of other neuro-ophthalmology specialists in the UK who are already organized in national organizations (UKNOSIG, BINOC), might be only beneficial, but I might ignore local barriers, of course.

- Can the authors describe the way they choose the participants, in order to be representative? Are the most recognized neuro-ophthalmologists in the country involved in this process?

ABSTRACT:

- I do not think that the Abstract should include the unfortunate story of the optometrist who got convicted for missed papilledema, this has been already largely described in the past. A factual Abstract might be preferred.

- I do not think that the title should be repeated in the Abstract, even as work programme

- I do not think that the "Ethics" paragraph should be part of the Abstract, this is quite intuitive

- The final paragraph on "strenghts and limitations" is excellent. I would just remove the word "robust"

MEHODS

- Can the authors provide a plan of the proposed questions and the clinical situations described in the clinical vignettes? In particular, some situations may be subtle, yet critical, i.e. papilledema in children (and what are the normative values on OCT)? Is lumbar puncture mandatory in patients with suspected mild papilledema, etc.?

- OBJECTIVE1 - Concerning the semi-structured interviews, have the authors evaluated if 10 optometrists, 10 GPs and 10 ophthalmologists were sufficient to obtain a robust result? Why not include also neurologists, who are also supposed to manage papilledema detection, including evasluation of the retinal fundus?

Reviewer **2**

Name **Aghaji, Ada**

Affiliation **University of Nigeria Faculty of Medical Sciences, Department of Ophthalmology**

Date **16-Sep-2024**

COI **No competing interests.**

This is a topical issue, and the authors should be congratulated for undertaking this research. The rationale for the study is excellent. However, there are a few concerns.

1. The introduction is verbose and can be made more succinct.

2. Is it necessary to include the name of the optometrist that failed to diagnose papilloedema?

3. Under objective 2. Is the proposed DIPP for health practioners or for patients. This is not clear. If the proposed DIPP is to facilitate clinician diagnosis of pseudopapilloedema, will patients need to understand the proposed DIPP guidelines and why?

4. DIPP study guidelines have not been validated or approved so the word proposed should be used.

5. Methods 1.3. What is the additional benefit of doing a video interview if all the information can be captured on audio?

6. Methods 1.4. You state that suitable quantitative evidence will be summarised using descriptive statistics. What variables are you assessing?

7. Methods 2.1. Delphi process. You state that t"hese items will be derived from published literature." This is the first time a literature review is being mentioned. Has it been done already by the current authors? If so, provide a reference and mention the search terms and strategy. Or is it part of this current study? If so, show a complete search strategy.

8. You state that, "The surveys will be kept to a maximum time of 20 minutes to complete to avoid attrition"

You can only suggest that the surveys will take about 20 minutes as participants response times will vary.

9. Recruitment of participant groups is not clear. You state that you will advertise, contact relevant bodies and accept all that accept to participate. Then you state you will undertake purposive sampling. Purposive sampling is preferred to get experts' views. Please state how the purposive sampling will be conducted and the inclusion and exclusion criteria.

10. How will you ensure representation across the different participant groups- GPs, optoms and ophthalmologists, etc. and geographical spread?

11. You mention a steering group halfway through the process. It should have been mentioned earlier. Who are the members of this steering group? What are their qualifications. What is the role of the group? - Literature review? Development of Round 1 Delphi questionnaire? Please state.

12. Apart from ranking, what other questions will be asked in round 1? The Delphi questions should reflect the research questions associated with objective 2.

13. How will results of the Delphi be analysed. How will consensus be determined?

14. For objective 3, we may be jumping the gun. The proposed DIPP will need to be validated and approved by appropriate regulatory bodies before it can be rolled out.

15. It is a good study and the rational is excellent.

16. I suggest the authors read up more Delphi studies to get a fundamental understanding of how a Delphi exercise works.

17. Some of the references are out of date. There are more recent studies on the Delphi methodology.

VERSION 1 - AUTHOR RESPONSE

Reviewer: 1

Dr. Dan Milea, Singapore National Eye Centre, Singapore,

Comments to the Author:

In the proposed article, the authors describe very thoroughly their planned research on referrals for papilledema (and its differential diagnosis). There is a high need for such a study and the authors have produced a proposal which very well-conceived, most likely due to the collaborative participation of experts from multiple fields (GPs, ED doctors, ophthalmologists, neurologists, statisticians, population health experts, etc). I have only a few, very limited remarks

Response

Thank you for your supportive comments

I do not see the dates of the planned study in the manuscript

Response

As above in Editors comments section

This text has been added under study design within methods

'The study commenced May 2022 and has a planned end date of March 2025.'

Why is this study limited to England and does not include the whole UK, as other major national studies have done before? Having the participation of other neuro-ophthalmology specialists in the UK who are already organized in national organizations (UKNOSIG, BINOC), might be only beneficial, but I might ignore local barriers, of course.

Response

The National Health Service (NHS) is the umbrella term for the publicly funded healthcare systems of the United Kingdom (UK) comprising the NHS in England, NHS Scotland and NHS Wales. Health and Social Care in Northern Ireland was created separately and is often locally referred to as "the NHS". The original three systems are run independently financially with different albeit similar strategies and programmes. Thus, our study focuses on the NHS in England which even within this region there is much variation in provision of services. That said, we aim for our guidance to be broadly applicable to the regions of the UK.

Can the authors describe the way they choose the participants, in order to be representative? Are the most recognized neuro-ophthalmologists in the country involved in this process?

Response

On page 9 we outline our recruitment strategy

‘These groups and networks will include the College of Optometrists, Royal College of Ophthalmologists, Royal College of Emergency Medicine, National Institute of Health and Care Research (NIHR) Clinical Research Networks (CRNs), Association of British Neurologists and UK Neuro-ophthalmology special interest group to ensure representation of eye care professions. We will not be requesting mailing lists but will ask these professional groups and networks to send out a recruitment email on our behalf. We will also recruit via our local National Institute of Health and Care Research (NIHR) Clinical Research Networks (CRNs) who can reach out to the other national CRNs. This will allow to recruit general practitioners.’

We are reliant on professionals wanting to be part of the study but we believe that working with these professional bodies is the best way to capture a range of professionals and experts in the fields.

ABSTRACT:

- I do not think that the Abstract should include the unfortunate story of the optometrist who got convicted for missed papilledema, this has been already largely described in the past. A factual Abstract might be preferred.

- I do not think that the title should be repeated in the Abstract, even as work programme

- I do not think that the "Ethics" paragraph should be part of the Abstract, this is quite intuitive (****editor’s note: please follow our Instructions for Authors which require an Ethics and Dissemination section in the abstract (and the main text).)

- The final paragraph on "strengths and limitations" is excellent. I would just remove the word "robust"

Response

We have removed both the optometrist story, and the title of the programme as requested. We have kept in the ethics as required by the journal .

We have edited the strengths and limitations statements as requested by the Journal and have replaced the word ‘robust’ with ‘established’

MEHODS

- Can the authors provide a plan of the proposed questions, and the clinical situations described in the clinical vignettes? In particular, some situations may be subtle, yet critical, i.e. papilledema in children (and what are the normative values on OCT)? Is lumbar puncture mandatory in patients with suspected mild papilledema, etc.?

Response

We have added the two vignettes to appendices (Appendix 1). We have flagged this up in the text (p7)

OBJECTIVE1 - Concerning the semi-structured interviews, have the authors evaluated if 10 optometrists, 10 GPs and 10 ophthalmologists were sufficient to obtain a robust result? Why not include also neurologists, who are also supposed to manage papilledema detection, including evaluation of the retinal fundus?

Response

The purpose of the SS interviews is to add real life experience and context to the semi-quantitative data of the surveys as opposed to a bona fide qualitative study although we have used established qualitative methods, we are not working towards data saturation with our interviews (Saunders 2018 doi: 10.1007/s11135-017-0574-8) . We have clarified this in the text.

We have not included neurologists in the interview schedule as we felt they are at the specialist end of the pathway and interviews would not provide us with sufficient relevant information for our guidance. We have clarified this in the text. However, we did feel it was of value to survey them to provide the reference point within secondary care.

Reviewer: 2

Dr. Ada Aghaji, University of Nigeria Faculty of Medical Sciences, London School of Hygiene and Tropical Medicine International Centre for Eye Health

Comments to the Author:

This is a topical issue, and the authors should be congratulated for undertaking this research. The rationale for the study is excellent. However, there are a few concerns.

Response

Thank you for your support

1. The introduction is verbose and can be made more succinct.
   Is it necessary to include the name of the optometrist that failed to diagnose papilloedema?

Response

We have removed the name and detail of the Honey Rose case and made modest reduction of other text but overall feel that the introduction is warranted to contextualise our objectives and underling research questions.

2. Under objective 2. Is the proposed DIPP for health practitioners or for patients. This is not clear. If the proposed DIPP is to facilitate clinician diagnosis of pseudopapilloedema, will patients need to understand the proposed DIPP guidelines and why?

Response

The guidance is for diagnosis and pathway is aimed at health professionals but as part of that we are producing patient friendly information (probably leaflets) that health professionals will be able to use to discuss care and the pathway with the patients.

We have taken out the words 'for patients' from the last research question for clarity. The generation of patient friendly information is covered by objective 3 which we have edited to make it clear what we are producing.

3. DIPP study guidelines have not been validated or approved so the word proposed should be used.

Response

Agree, we have put proposed guidelines in objective 3.

4. Methods 1.3. What is the additional benefit of doing a video interview if all the information can be captured on audio?

Response

The aim is to facilitate an in person interview setting – to enhance rapport between the interviewer and the interviewee. However as see we have given the participants the options of how they would like to speak to us.

Response

5. Methods 1.4. You state that suitable quantitative evidence will be summarised using descriptive statistics. What variables are you assessing?

Response

We have put the detail of these questions in Appendix 2, and flagged it up in the text.

Quantitative evidence from the survey questions regarding the case -vignettes (e.g. diagnosis and referral destinations) and information about current practice, for example, availability of local protocols, and referral trends will be summarised using descriptive statistics

6. Methods 2.1. Delphi process. You state that these items will be derived from published literature." This is the first time a literature review is being mentioned. Has it been done already by the current authors? If so, provide a reference and mention the search terms and strategy. Or is it part of this current study? If so, show a complete search strategy.

Response

Yes, there is corresponding literature review. It is currently awaiting peer review. We have cited the Prospero protocol PROSPERO [CRD42020213223]. This protocol includes the search strategy. We have added this information to the text.

You state that, "The surveys will be kept to a maximum time of 20 minutes to complete to avoid attrition "You can only suggest that the surveys will take about 20 minutes as participants response times will vary.

Response

Agree, we have changed the text 'The surveys are designed to take a suggested time of 20 minutes to complete to avoid attrition between survey rounds.'

7. Recruitment of participant groups is not clear. You state that you will advertise, contact relevant bodies and accept all that accept to participate. Then you state you will undertake purposive sampling. Purposive sampling is preferred to get experts' views. Please state how the purposive sampling will be conducted and the inclusion and exclusion criteria.

   How will you ensure representation across the different participant groups- GPs, optoms and ophthalmologists, etc. and geographical spread?

Response

We have added in extra detail to the recruitment text to explain how we will get geographical and professional representation. (P8) We have edited the description of purposive sampling for the Delphi participants in the relevant section (p11)

We will use purposive sampling to ensure we capture the expert opinions of health professionals from across England, by inviting those with a reputation/publication record in this area through the support of our research team who have expertise in these specialities and contacts within national networks and professional bodies.

11. You mention a steering group halfway through the process. It should have been mentioned earlier. Who are the members of this steering group? What are their qualifications. What is the role of the group? - Literature review? Development of Round 1 Delphi questionnaire? Please state.

Response

We have put the details of our steering group for the DIPP study in the methods. These collaborators and advisors were brought on board from the start of the primary research. The systematic project was standalone project both time-wise and funding-wise. To fit in with title and contributing statements we have renamed this group 'the working group'.

8. Apart from ranking, what other questions will be asked in round 1? The Delphi questions should reflect the research questions associated with objective 2.

Response

There are no other questions, what we have described on page 13 addresses objective 2.

Objective 2: *To reach consensus among health professional experts, commissioners, and patients on what the most evidence-based and safe diagnostic and referral practices should be for people with papilloedema/pseudopapilloedema across England.*

The focus in Round 1 is to identify the important elements which differentiate papilloedema from pseudopapilloedema – this addresses the diagnostic elements. It is necessary to form consensus on diagnosis before we can draw consensus on the referral process. Building on what the consensus diagnostic elements are, the focus in subsequent rounds/consensus meeting will address the referral practices/process.

9. How will results of the Delphi be analysed. How will consensus be determined?

Response

We have added this sentence: Data will be exported from REDCap for descriptive analysis in Microsoft Excel (e.g. median, percentages). Free text responses will be narrative summarised. Elements that participants propose as important for consideration that were not previously included, will be surveyed in the next stage of the process (p13). How consensus will be determined in the virtual meeting and the definition of consensus was provided on page 14. The consensus definition now follows this new sentence.

For objective 3, we may be jumping the gun. The proposed DIPP will need to be validated and approved by appropriate regulatory bodies before it can be rolled out. We have edited objective 3 (In both places) to reflect we will be at start of this process at the end of our study,

It is a good study and the rational is excellent.

Response

Thank you

I suggest the authors read up more Delphi studies to get a fundamental understanding of how a Delphi exercise works.

Some of the references are out of date. There are more recent studies on the Delphi methodology.

Response

Thank you we take this on board and will continue to educate ourselves as we proceed. However, we do not consider our methodology as proposed to be inappropriate.

VERSION 2 - REVIEW

Reviewer 2

Name Aghaji, Ada

Affiliation University of Nigeria Faculty of Medical Sciences, Department of Ophthalmology

Date 20-Nov-2024

COI

The authors have addressed the comments satisfactorily. However, there are a few minor edits.

1. What are the limitations of a Delphi study? This should be clearly spelt out. It provides the lowest level of evidence as it is based on expert opinion as compared with an RCT.

2. PPIE should be spelt out in full when introduced.

3. Talking about English services. The word English is ambiguous and should read services in England.

4. For Objective 1, What response rates are you targeting and what steps are you taking to achieve this?

VERSION 2 - AUTHOR RESPONSE

Comments to the Author:

The authors have addressed the comments satisfactorily. However, there are a few minor edits.

1. **What are the limitations of a Delphi study? This should be clearly spelt out. It provides the lowest level of evidence as it is based on expert opinion as compared with an RCT.**

The Delphi process is an appropriate methodology for our overall aim to write clinical guidelines and educational materials that will help support health professionals to correctly identify people with papilloedema. As part of the Delphi process, we are using the most up to date international evidence including the results of published systematic reviews and meta-analysis, contextualised within current service provision in the England region, that we have summarised to present to relevant experts from different health professions- ultimately to produce UK relevant (acknowledging regional variation) clinical guidance to all health professionals involved, as well as supportive literature and guidance for the public. As such, the DIPP Delphi study does not rely purely on expert opinion to generate findings (a common criticism). We are also clear on how we measure consensus which can also be a criticism. It is a flexible approach but of course it has its limitations – participants may change their opinions between rounds and there may be considerable dropouts between rounds as well. **We have added some text into our methods to cover these points.**

A RCT approach produces one of the highest levels of evidence for the efficacy of a medical intervention. Once we have written the DIPP clinical guidelines, our aim is to test their efficacy in improving the diagnostic accuracy of referrals for papilloedema. An RCT may be appropriate for this purpose, if we consider a comparison between sites that have implemented our guidance with those that have not. However, it more likely that we would take a pragmatic before and after approach with such an intervention.

2. **PPIE should be spelt out in full when introduced.**

We have revised the manuscript as we realise, we have used both the terms PPI and PPIE for the same groups. Patient and Public Involvement and Engagement (PPIE) is currently the most accepted term, so we have consistently used this term throughout the manuscript now. We have used descriptive text to describe our patient and public members in the strengths and limitations statements.

3. **Talking about English services. The word English is ambiguous and should read services in England.**

Agree, we have corrected this when present.

4. **For Objective 1, What response rates are you targeting and what steps are you taking to achieve this?**

For the FOI requests to ICBs we are expecting a 100% response, as it is a legal requirement for ICBs to respond with 20 days – **we have added some text to emphasise that.**

We are not able to define a response rate for the surveys as we cannot enumerate the population we will reach through the networks and mailing lists of the organisations we are working with to disseminate the surveys, as they are kept strictly confidential. Our approach is

to be inclusive and get good regional variation in the survey responses. **We have added some text to explain that.**

We have stated we will interview up to 10 participants from each health professional group – we will stop conducting interviews when we feel we have reached saturation of ideas**. We have added some text to explain that too.**
